# Carbonate Apatite Containing Statin Enhances Bone Formation in Healing Incisal Extraction Sockets in Rats

**DOI:** 10.3390/ma11071201

**Published:** 2018-07-12

**Authors:** Yunia Dwi Rakhmatia, Yasunori Ayukawa, Akihiro Furuhashi, Kiyoshi Koyano

**Affiliations:** Section of Implant and Rehabilitative Dentistry, Division of Oral Rehabilitation, Faculty of Dental Science, Kyushu University, 3-1-1 Maidashi, Higashi-ku, Fukuoka 812-8582, Japan; ayukawa@dent.kyushu-u.ac.jp (Y.A.); furuhasi@dent.kyushu-u.ac.jp (A.F.); koyano@dent.kyushu-u.ac.jp (K.K.)

**Keywords:** carbonate apatite, bone substitute, micro-CT, rat mandibular incisor, tooth extraction

## Abstract

The purpose of this study was to evaluate the feasibility of using apatite blocks fabricated by a dissolution–precipitation reaction of preset gypsum, with or without statin, to enhance bone formation during socket healing after tooth extraction. Preset gypsum blocks were immersed in a Na_3_PO_4_ aqueous solution to make hydroxyapatite (HA) low crystalline and HA containing statin (HAFS), or in a mixed solution of Na_2_HPO_4_ and NaHCO_3_ to make carbonate apatite (CO) and CO containing statin (COFS). The right mandibular incisors of four-week-old male Wistar rats were extracted and the sockets were filled with one of the bone substitutes or left untreated as a control (C). The animals were sacrificed at two and four weeks. Areas in the healing socket were evaluated by micro-computed tomography (micro-CT) and histological analyses. The bone volume, trabecular thickness, and trabecular separation were greatest in the COFS group, followed by the CO, HAFS, HA, and C groups. The bone mineral density of the COFS group was greater than that of the other groups when evaluated in the vertical plane. The results of this study suggest that COFS not only allowed, but also promoted, bone healing in the socket. This finding could be applicable for alveolar bone preservation after tooth extraction.

## 1. Introduction

Adequate bone volume and bone density are prerequisites for a predictable long-term prognosis in implant dentistry. Insufficient horizontal or vertical bone in patients precludes the successful outcome of an ideal implant placement [1]. Additional materials, such as autografts, allografts, xenografts, or synthetic bone substitutes are often required to increase and augment the bone volume. In recent years, researchers have developed and fabricated synthetic bone substitutes to achieve a high relative amount of new bone, while avoiding or minimizing the risks of the invasive harvesting of bone from a healthy site, disease transmission, and antigenicity [2].

Calcium sulfate dihydrate (CaSO_4_·2H_2_O), known as gypsum, has been approved by the U.S. Food and Drug Administration for clinical use to reconstruct bone defects [3]. Gypsum has the ability to undergo in situ setting after filling the defect, has good biocompatibility, and promotes bone healing [4]. In addition, gypsum can be produced by mixing CaSO_4_·0.5H_2_O powder and water. It is self-setting and can be molded and shaped at room temperature. Gypsum is slightly soluble in water and is thermodynamically unstable in a phosphate-salt-containing solution. It has also been reported that gypsum immersed in a sodium phosphate solution can be transformed to hydroxyapatite [5].

Hydroxyapatite [HA, Ca_10_(PO_4_)_6_(OH)_2_] is considered to be a promising bone substitute in the orthopedic and dental fields because of its high biocompatibility and osteoconductivity [6]. Most HA products are prepared by sintering chemically prepared HA powder at a high temperature. Although the sintering of HA powder provides monolithic HA with good mechanical strength, the crystallinity of the product is too high to be reabsorbed by osteoclasts [7]. To improve this shortcoming, a new method has been proposed to fabricate low-crystalline, porous hydroxyapatite blocks treated with trisodium phosphate solution, using a compositional transformation reaction based on a dissolution–precipitation reaction, with preset gypsum as a precursor [8]. 

The inorganic component of bone consists of hydroxyapatite with an apatitic crystal solid structure, and contains impurities [9]. The most common impurity is carbonate, which replaces 4–8% of the phosphate groups [10]. In terms of chemical composition, the inorganic component is a carbonated, basic calcium phosphate; hence, it can be termed a carbonate apatite (CO_3_Ap: Ca_10_-_a_(PO_4_)_6_-_b_(CO)_c_(OH)_2_-_d_) [11,12]. Sintering is not suitable for the fabrication of CO_3_Ap blocks because of the low thermal stability of CO_3_Ap at high temperatures, >400 °C [13]. Therefore, a method was proposed to fabricate CO_3_Ap blocks by a dissolution–precipitation reaction, with a preset gypsum as an artificially fabricated precursor. Previous studies have described the fabrication on the treatment of preset gypsum with carbonate ion sources added into the system [14,15]. The gypsum blocks were immersed in a mixture of 0.4 mol/L disodium hydrogen phosphate (Na_2_HPO_4_) and 0.4 mol/L Sodium hydrogen carbonate (NaHCO_3_) [14]. Sodium hydrogen carbonate and disodium hydrogen phosphate were used as supply sources of CO_3_^2−^ and PO_4_^3−^ ions [10]. However, another previous study reported that the immersion of preset gypsum in a sodium phosphate solution also produces carbonate apatite, although the carbonate ions are supplied from the atmosphere as CO_2_, particularly when the phosphate salt solution is alkaline [16]. The gypsum used as the precursor should have low solubility and must not disintegrate in the solution to allow a balanced dissolution and precipitation process [14]. The fabrication of CO_3_Ap blocks in this manner is thought to be a promising artificial bone substitute that mimics bone in terms of chemical inorganic composition.

The mechanism of action of the materials used for bone regeneration is osteoconduction, which provides a scaffold for enhanced bone tissue growth and formation. A promising technique to increase the bioactivity of carbonate apatite blocks is the addition of osteoinductive growth factors or drugs incorporated into the composite. Statins are cholesterol-lowering drugs that inhibit 3-hydroxy-3-methylglutaryl-coenzyme A (HMG-CoA) reductase. A study reported that statin stimulated the bone morphogenetic protein (BMP)-2 expression and showed positive effects on bone formation [17]. Statins have been widely used in alveolar ridge augmentation and bone grafting in the craniofacial region, because of their osteoinductive effect [18,19,20]. Previous studies reported that the systemic administration of simvastatin promoted bone formation around implants [21] and a topical application of fluvastatin led to bone formation around tibial titanium implants [22]. In addition, the injection of poly(lactic-co-glycolic) acid PLGA-fluvastatin microspheres promoted both bone formation and gingival soft tissue healing [23,24]. Jinno et al. reported that atelo-collagen and alpha-tricalcium phosphate (α-TCP) as a carrier successfully promoted vertical bone formation on the parietal region [25]. Additionally, solutions of statin in optimal concentrations could be combined with bone grafts to enhance their regenerative potential [26,27]. A recent study reported that statin also had antibacterial, antiviral, and antifungal effects that could alter its advantages in clinical dentistry [28].

Dental implant treatment is usually associated with tooth extraction. Bone healing after tooth extraction may prolong the treatment period of 3–6 months. To shorten the treatment period, the preservation of sufficient bone volume and the early healing of alveolar bone following implant placement are desirable. The purpose of the present study was to investigate the effect of statin-containing carbonate apatite and to assess the amount of bone formation induced after the application of this composite in rat incisor extraction sockets.

## 2. Materials and Methods 

### 2.1. Preparation of Specimens 

Commercially available calcium sulfate hemihydrate (CaSO_4_·0.5H_2_O, Wako Pure Chemical Industries, Osaka, Japan) was mixed with distilled water at a water to powder ratio of 1:2. For the fluvastatin (FS) group, 0.5 mg FS (Toronto Research Chemicals, North York, Ontario, Canada) was added and mixed with 1 g calcium sulfate hemihydrate paste. The paste was packed into a cylindrical stainless steel mold (6 mm in diameter and 3 mm thick). Both sides of the mold were covered with glass plates and kept at room temperature for 24 h to set the gypsum. The preset gypsum block was then crushed and sieved to obtain 200–400 μm granules. 

To make low crystalline apatite, six gypsum granules without FS (HA group) or containing FS (HAFS group) were placed in each vessel (Shikoku Rika, Kochi, Japan) for hydrothermal treatment and immersion in 15 mL of 1 mol/L trisodium phosphate (Na_3_PO_4_, Wako) aqueous solution, as described previously [8]. The vessels were then placed in an oven (DO.300; As One, Osaka, Japan) at 100 °C for 24 h. 

To make the carbonate apatite specimens, the preset gypsum granules without FS (CO group) or containing FS (COFS group) were treated with phosphate and carbonate solution, as described previously [14]. About six gypsum granules from each group were immersed in a 15 mL mixture of 0.4 mol/L disodium hydrogen phosphate (Na_2_HPO_4_, Wako) and 0.4 mol/L sodium hydrogen carbonate (NaHCO_3_, Wako), placed in a hydrothermal vessel, and kept at 200 °C for 24 h in a drying oven. After the treatment, the specimens were washed with distilled water and dried at 60 °C for 24 h. The specimen preparation is summarized in Table 1.

### 2.2. X-Ray Diffraction Analysis 

The specimens were ground to a fine powder and the composition and crystallite size were characterized by X-ray diffraction (XRD) analysis. The XRD patterns were recorded using a powder X-ray diffractometer (D8 Advance A25, Bruker AXS GmbH, Karlsruhe, Germany) with CuKα radiation, operated at a tube voltage of 40 kV and a tube current of 40 mA. 

### 2.3. Scanning Electron Microscope Analysis

The fractured surfaces of the specimens were morphologically evaluated using a scanning electron microscope (SEM; S-3400N, Hitachi High-Technologies, Tokyo, Japan) at an accelerating voltage of 10 kV, after coating with gold-palladium. 

### 2.4. Animals

There were 48 four-week-old male rats that were used in this study; they were fed a commercially-available standard rodent food (CE-2, CLEA Japan, Tokyo, Japan). Water was available ad libitum. The protocol for this study was approved by the Animal Care and Use Committee of Kyushu University (approval number: A-26-064-0).

### 2.5. Anesthesia and Surgical Procedures

**Figure 1:**
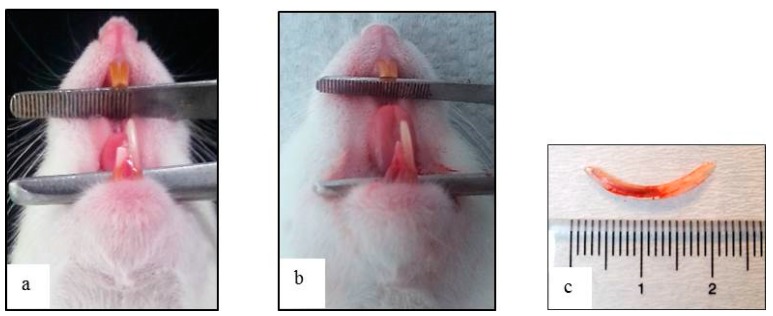
(**a**) Intraoral view after the crown of the mandibular right incisor was cut at the gingival level at 3, 6, and 9 days prior to extraction; (**b**) extraction of the lower right incisor; and (**c**) extracted incisor displaying no signs of fracture.

The crown of the mandibular right incisor was cut at the level of the marginal gingiva using a diamond disk with a micromotor handpiece, under anesthesia, every three days prior to extraction so as to loosen the retention by the periodontal ligament and to facilitate the tooth extraction. On the third of the three day periods, the incisor was carefully extracted in a horizontal direction along the long axis of the incisor, under general anesthesia (Figure 1).

In the experimental group, the extracted sockets were filled with 60 mg of either HA, HAFS, CO, or COFS, which was condensed with a root canal plugger using a controlled light force. The sockets were filled to 1 mm short of the orifice in order to avoid infection. In the control (C) group, the sockets were left untreated. At two and four weeks after the incisor extraction and specimen implantation, the animals were deeply anesthetized and perfused with a fixative solution consisting of 0.1 M phosphate-buffered 4% paraformaldehyde (pH 7.4). For a micro-computed tomography (micro-CT) and histological analysis, the right mandibles without soft tissue were dissected out and the samples were fixed in 10% formalin for one week.

### 2.6. Micro-Computed Tomography Analysis

Unprocessed mandibles were imaged and analyzed using an in vivo micro-CT scanner (SkyScan 1076, Aartselaar, Belgium) at 60 kV/167 μA and an Al-0.5 filter. The specimens were fitted into a cylindrical sample holder and scanned in horizontal and vertical positions. High-resolution scanning with a slice thickness of 18 μm was performed. For the micro-CT analysis, a region of interest (ROI) was determined so as to evaluate the socket bone healing in both the horizontal and vertical planes. 

The ROI analysis was performed to assess the primary parameters of the bone volume (BV) and the total tissue volume (TV), both measured in mm^3^. The TV is the volume of the whole examined sample. This volume is typically defined by a contour or mask, which includes the volume of interest (VOI). The BV was calculated as the volume of the region characterized as bone and normalized ratiometrically against the total volume of the region of interest (BV/TV), in order to derive the percentage bone ratio (%). Bone with different degrees of mineralization (bone mineral density [BMD]) (g/cm^3^) records different densities and linear attenuation coefficients, resulting in gray-value variations in the CT scans. Other parameters were trabecular thickness (Tb.Th) to measure the thickness of bone trabeculae (1/mm) and trabecular separation (Tb.Sp) to measure the width of the gap between the bone trabeculae (1/mm).

**Figure 2:**
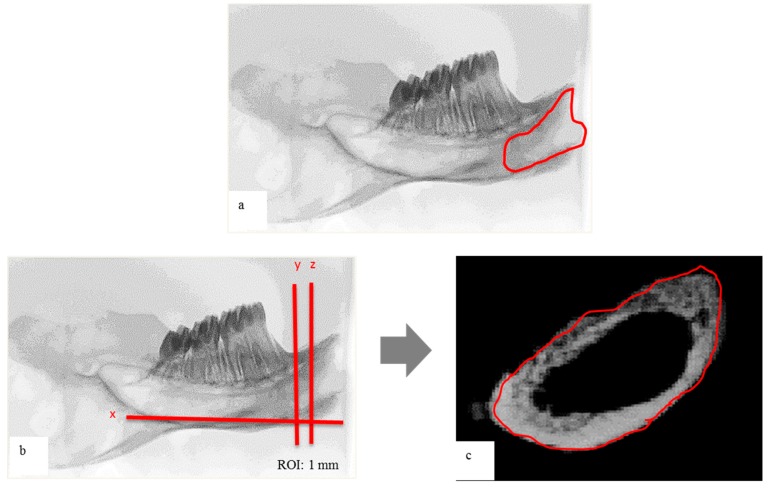
Micro-computed tomography (micro-CT) analysis: (**a**) radiographic image in the horizontal plane; (**b**) radiographic image in the vertical plane (x: mandibular plane, y: plane y; z: plane z), region of interest (ROI) was determined as 1 mm of bone thickness between y and z; (**c**) reconstructed image of ROI before analysis.

For the horizontal plane evaluation, the ROI was determined by interpolating the radiographic image on the socket area. For the vertical plane evaluation, the micro-CT scanner software (Version 1.10, Bruker/Skyscan μCT, Kartuizersweg, Kontich, Belgium) was used to make a three-dimensional (3-D) reconstruction from each set of scans. From the entire 3-D data set, an interpolated ROI of the vertical plane was determined, as described previously (Figure 2) [29]. The area of a thickness of 1 mm between the following two planes was observed: the first plane, which was vertical to mandibular plane (plane x), and tangential to the proximal border of the mandibular first molar (plane y), and the second plane, which was parallel and 1 mm medial to the first plane (plane z).

### 2.7. Histological Evaluation

Following the micro-CT scanning, the samples were dehydrated with a graded series of ethanol and were embedded in methacrylate resin. Undecalcified sagittal sections (thickness ~70 μm) were cut, polished, and stained using Masson’s trichrome method. For the histological evaluation of the bone and cellular tissue responses, the samples were examined under a light microscope. The center of the test material from one histological section of each specimen was selected to represent that group for evaluation. 

### 2.8. Statistical Analysis

The experimental data were assessed by analysis of variance (ANOVA) with Tukey–Kramer tests for post hoc analysis. The significance level was set at *p* <0.05. All of the statistical analysis was performed using SPSS 12.0 J (SPSS Japan, Tokyo, Japan).

## 3. Results

### 3.1. X-Ray Diffraction Analysis

**Figure 3:**
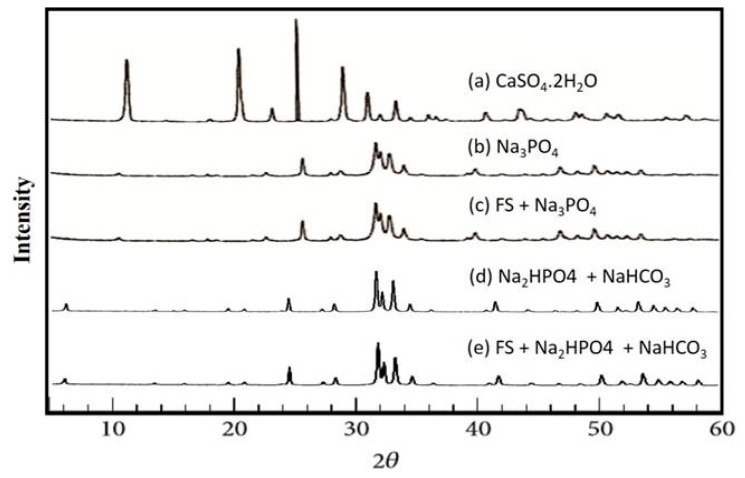
Powder XRD patterns of (**a**) preset gypsum before treatment; (**b**) preset gypsum after immersion in 1 mol/L Na_3_PO_4_ solution at 100 °C for 24 h; (**c**) preset gypsum containing fluvastatin after immersion in 1 mol/L Na_2_HPO_4_; (**d**) preset gypsum after immersion in 1 mol/L Na_2_HPO_4_ and 1 mol/L NaHCO_3_ at 200 °C for 48 h; and (**e**) preset gypsum containing fluvastatin after immersion in 1 mol/L Na_2_HPO_4_ and 1 mol/L NaHCO_3_ at 200 °C for 48 h.

The XRD patterns of the powdered granules used in this study are summarized in Figure 3. The preset gypsum granules were found to be CaSO_4_·2H_2_O (Figure 3a), and the preset gypsum immersed in Na_3_PO_4_ solution demonstrated a broad apatitic peak, indicating that it had undergone a compositional transformation from gypsum to low-crystalline apatite (Figure 3b). Similarly, the preset gypsum containing fluvastatin immersed in a Na_3_PO_4_ solution also demonstrated a broad apatitic peak (Figure 3c). 

The preset gypsum with or without fluvastatin and immersed in a solution of Na_2_HPO_4_ and NaHCO_3_ (Figure 3d,e) at 200 °C for 48 h also demonstrated a broad apatitic peak. Furthermore, an energy dispersive X-ray spectroscopy analysis of all of the specimens clarified that they did not contain any element except calcium and phosphor, and the X-ray diffraction analysis proved that all of the groups consisted of HA.

### 3.2. Scanning Electron Microscope Analysis

**Figure 4:**
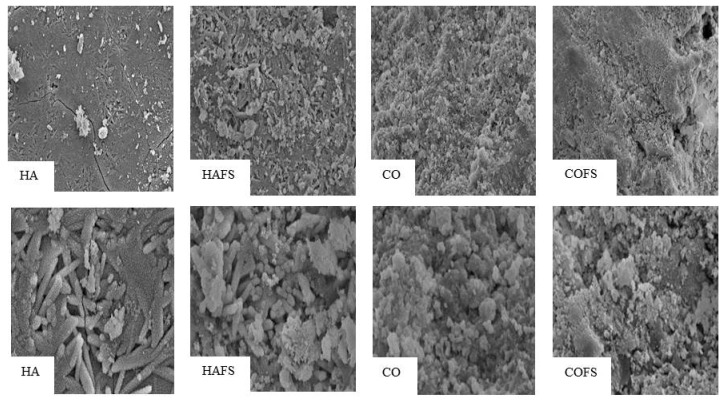
SEM analysis of all groups at magnification ×1000 (upper) and ×4000 (lower).

SEM images of HA, HAFS, CO, and COFS granules are shown in Figure 4. The density was somewhat higher in the HAFS granules than in the other granules. The HAFS granules retained the morphology of a needle-like gypsum crystal structure covered with many fine granular crystals. The morphology of the CO and COFS granules consisted of tight tangles of needle-like crystals that were smaller and less tangled than those of the HA and HAFS groups.

### 3.3. Micro-Computed Tomography Analysis

**Figure 5:**
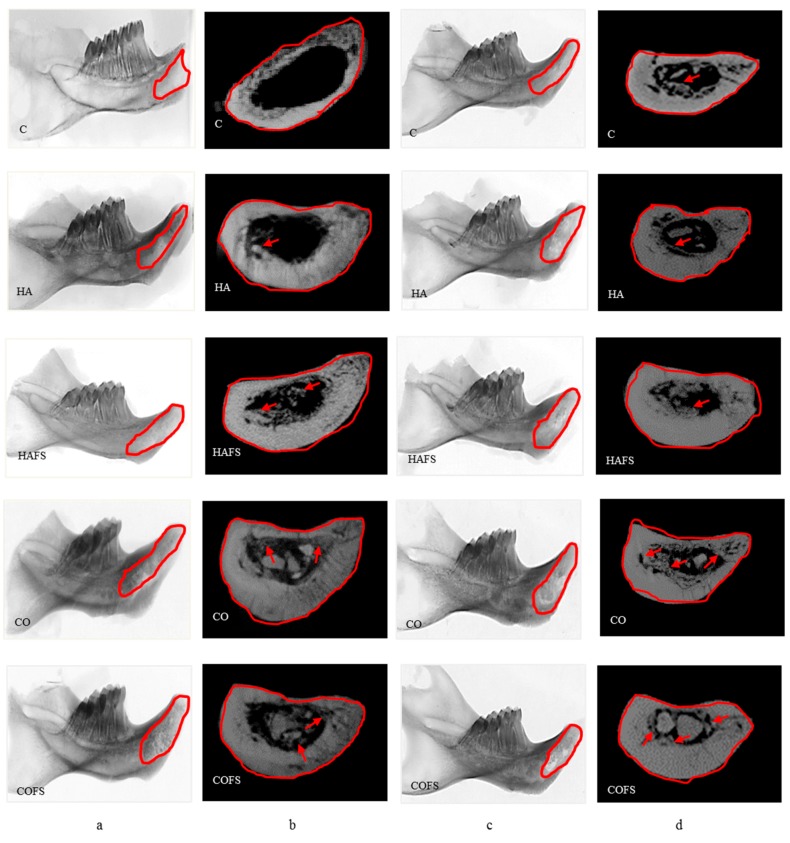
Micro-CT images of the lower right incisor extraction sockets: (**a**) in the horizontal plane at two weeks; (**b**) in the vertical plane at two weeks; (**c**) in the horizontal plane at four weeks; and (**d**) in the vertical plane at four weeks.

Micro-CT images of all of the groups in the horizontal and vertical planes at two and four weeks after extraction are shown in Figure 5. The micro-CT reconstruction in the vertical plane shows that the most bone formation occurred in the COFS and CO groups, followed by the HAFS and HA groups. More bone formation was observed in the four week groups than the two week groups. Bone growth was observed in the socket area of all of the groups; however, the bone surrounding the socket was thicker in the CO and COFS groups than in the other groups. This result indicates that the carbonate apatite did not hinder the natural bone healing process, but rather enhanced new bone formation in the socket area and the surrounding bone. 

**Figure 6:**
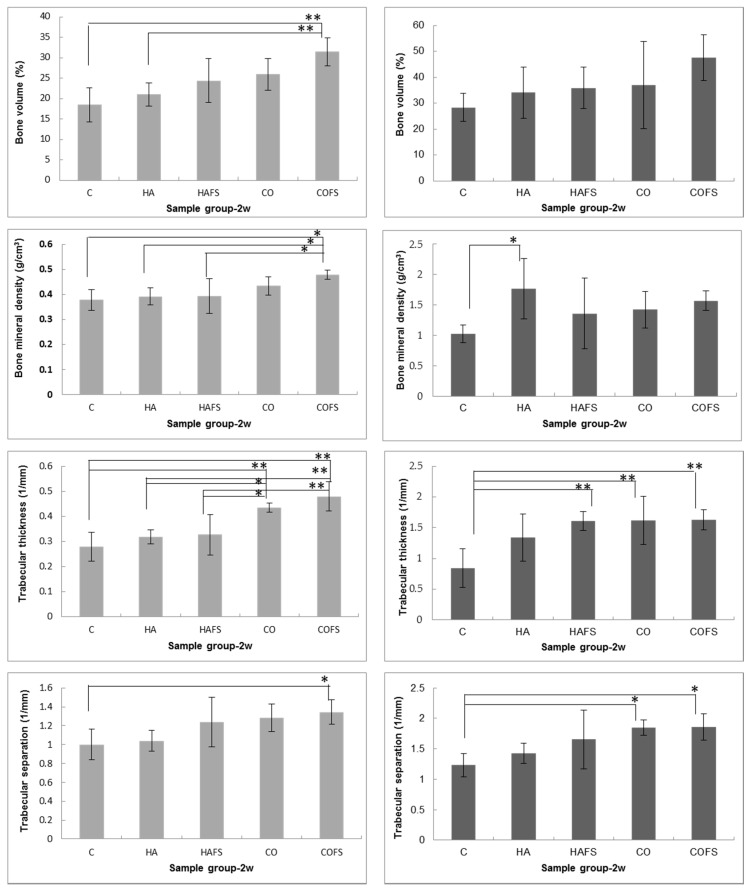

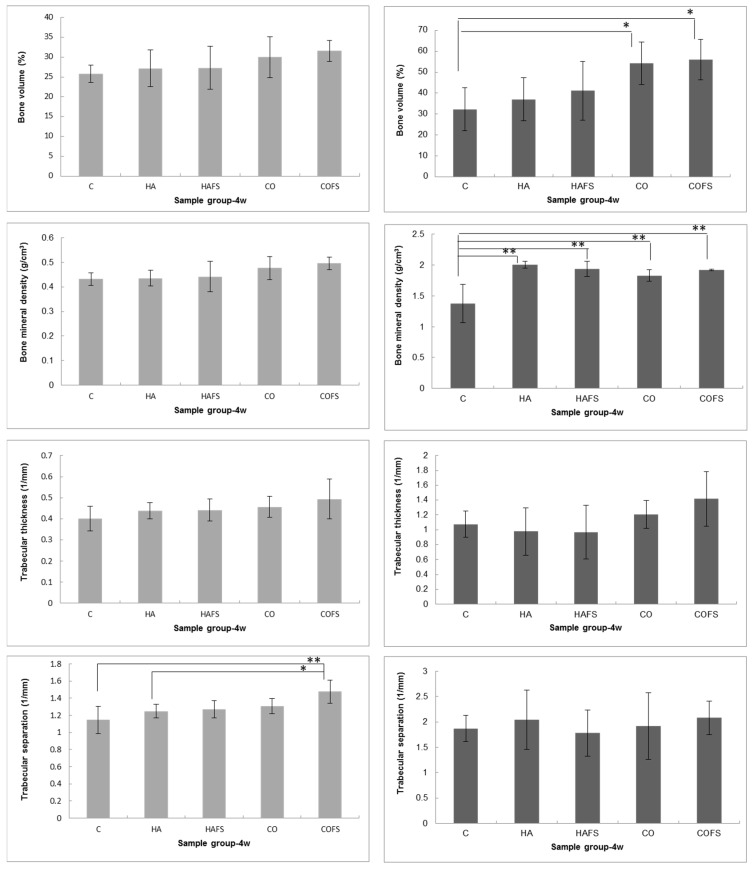
Micro-CT analysis of lower right incisor extraction sockets in vertical (left columns) and horizontal (right columns) planes at two and four weeks: bone volume, bone mineral density, trabecular thickness, and trabecular separation. A *p* value of <0.05 (*) and a *p* value of <0.01 (**) were considered significant.

The bone volume of the COFS group was greater than that of the other groups, both in the horizontal and vertical planes at two and four weeks after extraction. However, the BMD of the COFS group was lower than that of the HA group in the horizontal plane, but higher in the vertical plane. The difference in the ROI between the horizontal and vertical planes may have caused the differences in the bone volumes of the experimental groups. In addition, the values of Tb.Th and Tb.Sp tended to be higher in the experimental groups than in the control group. Notably, Tb.Th and Tb.Sp were significantly higher in the COFS group than in the CO, HAFS, HA, and control groups at two weeks (*p* < 0.05) (Figure 6).

### 3.4. Histological Evaluation 

**Figure 7:**
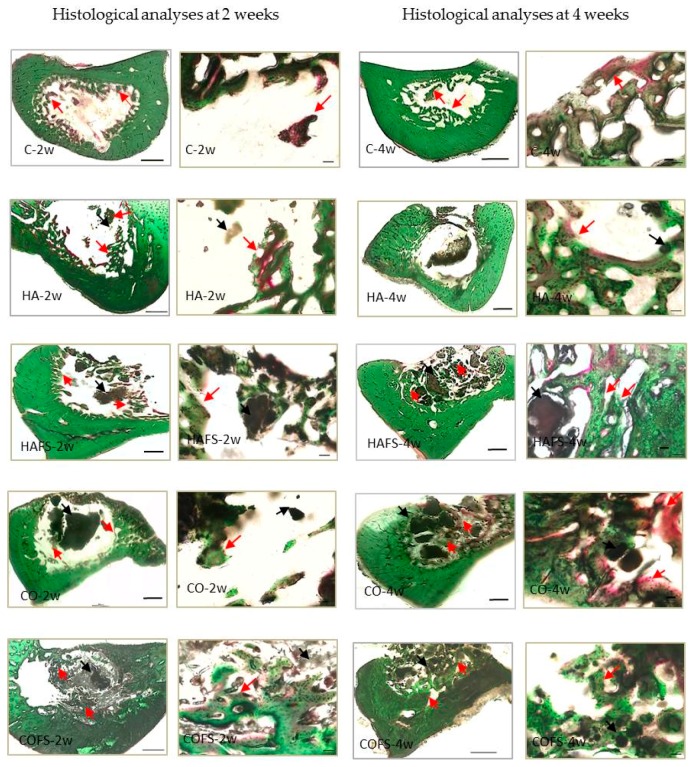
Histological images of lower right incisor extraction sockets at two and four weeks. Red arrows indicate new bone formation and black arrows indicate residual bone substitute in the healing socket. Magnification: ×4 Bar: 500 μm, ×20 Bar: 50 μm.

New bone formation was observed in all of the sample groups; however, a larger area of bone formation was observed in the COFS group, compared with the other groups, both at two and four weeks (Figure 7). Moreover, a higher mineralization density (as evidenced by the green staining) was observed in the COFS group when compared with the other groups. In the HA group, bone formation was observed in the form of red staining, which defines the lower mineralization density. The histological evaluation was in accordance with the micro-CT results in the vertical plane analysis, although the BMD in the horizontal analysis showed that the COFS group at four weeks tended to have a lower mineralization than the other groups.

## 4. Discussion

The prosthodontic treatment for the edentulous areas, including fixed or removable partial dentures and implants, is strongly related to the extraction socket and residual alveolar bone. The rapid healing potential of the extraction socket has generated interest, because it greatly influences subsequent dental treatment. A human study demonstrated that mineralization begins at the end of the first week, and that most of the granulation tissue has been replaced with a provisional matrix and immature bone by the sixth to eighth week of post-extraction healing [30]. However, because rat extraction sockets are considered to be a non-critical size defect, the socket will be gradually filled by newly-formed bone [29]. 

Various studies have explored the possibility of accelerating extraction socket healing and preserving the alveolar ridge [29,31]. A commonly used technique is bone grafting, using materials such as bovine bone mineral [32]; β-tricalcium phosphate [33]; or metabolic compounds, such as basic fibroblast growth factor [34] or simvastatin [35]. In this study, the rat sockets were treated with grafted bone as an osteoconductive material and fluvastatin as an osteoinductive material. The materials used to fill the socket area were low crystalline HA and CO_3_Ap, without fluvastatin (HA and CO) or with fluvastatin (HAFS and COFS). There were shortcomings of filling these materials in the rat incisor extraction sockets which were approximately 2 mm in diameter and 20 mm in depth; however, the micro-CT results from the experimental groups showed a similar bone formation pattern as the control group (C), indicating that the HA and CO_3_Ap, with or without fluvastatin, did not hinder the natural bone healing process in the socket. 

The micro-CT analysis was conducted in the horizontal plane to evaluate bone healing in the entire region of the extraction socket and in the vertical plane to establish the median extent of bone healing in the socket area. At two and four weeks, all of the BVs in the horizontal plane were higher than in the vertical plane, which confirmed that bone was formed throughout the whole socket area. The BVs in this study were higher at four weeks than at two weeks after the extraction. Similar previous studies reported that the initial bone formation in rat alveolar wound healing occurred at the end of the first week and that the bone mass increased gradually until the alveolar socket was totally filled with newly formed bone by the 21st day of the healing period [36,37]. However, recent studies reported that bone formation continued to proceed beyond 21 days post extraction, up to the sixth [38] or eighth [39] week. A fundamental step for the subsequent phases of bone healing in the socket area is the existence of blood clot formation [40]. In this study, the materials in the socket may limit the blood clot forming, and so delay the process of bone formation. A similar study reported that bone formation by the second week was delayed in the socket treated with inorganic bone relative to those treated with organic bone. The greater bone volume of the inorganic graft reached a similar amount to that which was observed in the animals grafted with organic bone by the ninth week [41]. The present study has indicated that grafted materials need to be resorbed, and thus, the new bone formation was delayed because of the limitation of blood supply and nutrients in the socket area. Therefore, the marked differences in methodology rendered comparisons between our results and those reported by Okamoto et al. [36] and Vieira et al. [37] unviable.

The BV of the COFS group was significantly higher than that of the other groups at four weeks, followed by the CO and HAFS groups. The results of this study demonstrated that the carbonate content in the COFS group was favored by the inorganic component of the original bone. Additionally, the statin mixed with CO_3_Ap and HA was observed to potentially enhance bone formation during socket healing. A previous study reported that there is evidence to suggest that statins, which have been safely used for treatment of hypercholesterolemia, enhance the biosynthesis of BMP-2 [42]. Other studies have reported the positive effects of statins on osteogenesis around implants and in tooth extraction sockets [23,24]. 

The HA group exhibited a lower level of bone formation. In contrast with this finding, a previous study found a higher level of bone formation with a hydrothermally-treated gypsum soaked in Na_3_PO_4_ solution than with sintered-HA granules in rat tibia after two weeks [16]. In the reaction to fabricate CO_3_Ap, the CO_3_^2−^ ions can be supplied in the form of CO_2_ from the atmosphere; however, in this study, the CO_3_^2−^ was added from a carbonate salt (NaHCO_3_) to the phosphate salt (Na_2_HPO_4_) solution. Thus, instead of transforming into CO_3_Ap, the hydrothermally-treated gypsum soaked in Na_3_PO_4_ transformed to low crystalline HA, resulting in a lower level of bone formation. The control group also exhibited minimal bone formation at two weeks, in contrast with a previous study, in which the normal untreated sockets exhibited progressive neo-bone-formation at two weeks [37]. In our preliminary in vitro study, the CO_3_Ap released calcium and phosphate ions that induced cell death and affected the osteoblastic activities. Other studies demonstrated that free calcium and inorganic phosphate ions influenced the osteogenic differentiation in vitro of osteoprogenitor cells. Therefore, they suggested making a clear link between the dissolution rate of the calcium phosphate in vitro and early bone formation in vivo [43]. Moreover, the features of the materials in vitro can affect the molecular and cellular interactions at their surface, and consequently can affect the process of bone formation. Differently, the interactions between the implant and its ‘biological surrounding’ in vivo are highly complex because of the non-equilibrium conditions and because of the undefined amount of compounds playing a role in these interactions [44].

The mineralization in the bone healing process was measured by comparing the BMD of the background bone with HA phantom rods, as part of the micro-CT analysis tools. The degree of mineralization, expressed in milligrams of HA phantom rods per cubic centimeter (mg HA/cm^3^), was found to be 0.25 to 0.75 mg HA/cm^3^. A threshold of 0.25 mg HA/cm^3^ was used to differentiate between the newly formed bone and background bone. Values above 0.75 mg HA/cm^3^ were assumed to be graft material. In the vertical plane evaluation at two weeks, the COFS group had a higher BMD than the other groups, but at four weeks, there was no significant difference among all of the groups. In the horizontal plane, the BMD was higher in the HA group than in the control and COFS groups. It is believed that the grafted bone acts as a mineral reservoir inducing bone formation via osteoconductive mechanisms [45]. The mineral content of the HA group was similar to that of the HA phantom rods, resulting in a higher BMD than that of the other groups. In contrast, a previous study using micro-CT reported that a greater bone volume appeared to be linked with lower bone density, probably because the rate of growth of the bone forming cells was greater than the rate of the bone mineralization [46].

The trabecular thickness (Tb.Th) was measured to compare the thickness of the trabecular structures. However, as Tb.Th is a scalar measurement, it may not be able to describe all of the structural changes. The trabecular separation (Tb.Sp) was determined as the mean distance between the mid-axes (i.e., the average separation between the mid-axes). The measurement of Tb.Th and Tb.Sp are related to the diameter of the interconnecting pores. The optimal diameter of the interconnecting pores allows the cells to attach and penetrate through the pores. If the diameter is too small, the cells have difficulty penetrating the structure and will only attach to the outside of the scaffold. In this study, the COFS group had higher Tb.Th and Tb.Sp scores compared with the other groups.

The histological sections of all of the groups supported the quantitative micro-CT findings. Newly-formed trabecular bone was observed mainly on the internal surfaces of the alveolar socket. New bone formation was observed on the surrounding bony walls, but was also seen in the form of ‘bony islands’ in the places where the socket was filled with grafted bone (HA, HAFS, CO, and COFS), while in the control group, new bone was formed only along the bony walls. Compared with the controls, the treated sockets seemed to contain a smaller amount of blood clot in association with larger amounts of connective tissue and bone. In the four week groups, the treated sockets had a greater relative volume of trabecular bone in parallel with a smaller amount of bone substitute when compared with the two week groups. This finding further supports the biocompatibility and osteoconductivity of HA and CO_3_Ap.

Previous studies have shown that the statin in calcium silicate/gypsum/gelatin composite has osteoinductive characteristics, which promote a higher level of bone formation when compared with the grafts without statin [47]. One study reported that the administration of statins increased the expression of bone morphogenetic protein (BMP)-2 mRNA, with a concomitant promotion of bone formation [42]. Statin, with a drug delivery system, has been reported to promote bone formation and soft tissue healing around implants, both through systemic administration or via topical application [21,22,23]. Statin has also been reported to enhance vascular endothelial growth factor (VEGF) production [48], and to exhibit antimicrobial effects [49]. Thus, statin has the potential to enhance bone formation in volume and quality. In this study, we also demonstrated that CO_3_Ap with statin supports a higher level of bone formation, and that it is effective as a bone substitute.

This study did not investigate the ability of the material to be fully absorbed. The bone grafts were observed to remain in the socket area, even after four weeks. However, during the bone remodeling process, osteoclasts produce a weak acidic environment in Howship’s lacunae at pH 3–5, to dissolve bone minerals. The solubility of apatite under weak acidic conditions increases with the carbonate content in its apatitic structure [50]. Therefore, CO_3_Ap is supposed to be resorbed by osteoclasts. On this premise, it is concluded that CO_3_Ap containing statin is an effective inorganic scaffold for bone regenerative therapy.

## 5. Conclusions

The present study suggests that gypsum as precursor can be transformed into hydroxyapatite and carbonate apatite. Materials containing statin were proven to be effective to promote bone formation. However, the present study should be regarded with caution because of its limitation of the physical and biological properties of carbonate apatite. Long-term evaluation is required to assess the clinical outcome. Another limitation of the present study was that it was difficult to fulfil the length and depth of the incisor socket area with our materials. Therefore, our data must be interpreted with caution. However, our findings suggest that the osteoconductivity function of CO_3_Ap containing fluvastatin promotes bone formation in the early stages of healing in extraction sockets. In light of the new developments in bone regeneration therapeutics, it is important to continue the development of low-cost biomaterials that involve minimal risk during treatment, and that are more reproducible.

## Figures and Tables

**Table 1:** Summary of preparation of all of the specimens. C—control; HA—hydroxyapatite low crystalline; HAFS—HA containing fluvastatin; CO—carbonate; COFS—CO containing FS.

Sample Groups	CaSO_4_·2H_2_O (Gypsum)	Statin	Immersion Solution	Hydrothermal Treatment
C	X	X	X	X
HA	O	X	Na_3_PO_4_	100 °C for 24 h
HAFS	O	O	Na_3_PO_4_	100 °C for 24 h
CO	O	X	Na_2_HPO_4_ and NaHCO_3_	200 °C for 24 h
COFS	O	O	Na_2_HPO_4_ and NaHCO_3_	200 °C for 24 h
